# Murine corneal stroma cells inhibit LPS-induced dendritic cell maturation partially through TGF-β_2_ secretion in vitro

**Published:** 2012-08-10

**Authors:** Jian-Min Lu, Xiu-Jun Song, Hui-Fang Wang, Xiao-Lei Li, Xiao-Rong Zhang

**Affiliations:** 1 Department of Ophthalmology, Third Hospital of Hebei Medical University, Shijiazhuang, China; 2 Department of Ophthalmology, First Affiliated Hospital of Dalian Medical University, Dalian, China

## Abstract

**Purpose:**

The peripheral cornea contains mature and immature resident dendritic cells (DCs) while the central cornea is exclusively equipped with immature DCs. There must be some factors that cause immature DCs. This study investigated whether corneal stroma cells (CSCs) inhibit DC maturation by secreting cytokines.

**Methods:**

The messenger ribonucleic acid (mRNA) and protein level of transforming growth factor beta 2 (TGF-β_2_) was analyzed using reverse transcription polymerase chain reaction (RT-PCR) and enzyme-linked immunosorbent assay (ELISA). Immature DCs were induced to mature in the presence of lipopolysaccharide (LPS) and with concentrations of CSC culture supernatant (containing and not containing neutralizing TGF-β_2_ antibodies). Then, the DC phenotypic and functional maturation were analyzed.

**Results:**

CSCs exhibited positive expressions of *TGF-β_2_* mRNA and secreted high concentrations of TGF-β_2_ protein. In the presence of LPS, DCs, which were treated with a CSC culture supernatant, displayed reduced expressions of cluster of differentiation 80 (CD80), CD86, and major histocompatibility complex II (MHC II) in a dose-dependent manner. Moreover, treated DCs showed lower T-cell stimulation capacity and a higher endocytosis function. However, these phenotypic and functional modifications were partially reversed after the application of neutralizing TGF-β_2_ antibodies.

**Conclusions:**

This study demonstrates that CSCs can partially inhibit LPS-induced DC maturation through TGF-β_2_ secretion in vitro.

## Introduction

Dendritic cells (DCs), the most efficient antigen-presenting cells (APCs), are rare but ubiquitously distributed in the mammalian body [1]. They are derived from cluster of differentiation 34+ (CD34^+^) bone marrow stem cells and can be generated from bone marrow hematopoietic progenitor cells in vitro through incubation with the granulocyte-macrophage colony stimulating factor (GM-CSF) and the tumor necrosis factor alpha (TNF-α) [2]. DCs, which possess the unique ability to stimulate naive T cells, play a key role in the production and delivery of antigens to different immune cells. Furthermore, the transition from antigen-processing cells to APCs is indispensable for initiating an immune response because immature DCs not only fail to prime T cells effectively, but they also serve to promote tolerance induction [3]. The differentiation and maturation process of DCs is associated with the upregulation of the expression of major histocompatibility complex class II (MHC II) and T-cell costimulatory molecules (CD80, CD86), enhancement of the T cell stimulation potential, and down-regulation of the antigen uptake ability [4].

Corneal transplantation (keratoplasty), which has high success rates, is the most commonly performed organ graft. From an immunologic point of view, a normal avascular cornea is thought to be an immune-privileged site without APCs. Moreover, previous studies that examined the cornea for APCs largely relied on expression of MHC II, which further strengthened this dogma [5]. However, this paradigm was shaken by Hamrah et al. [6,7], who found that the cornea was indeed endowed with a heterogeneous population of epithelial and stromal DCs, which function as APCs. Recently, Nakamura et al. [8] provided evidence of resident DCs in the cornea, showing that DCs were dispersed throughout the corneal stroma. Due to its direct exposure to the external environment, the cornea is frequently in contacts with exogenous pathogens. Accordingly, resident DCs of the cornea should be prone to maturation. However, previous studies showed a distinct distribution of DCs in the cornea; in the periphery of a normal cornea, many DCs were MHC II^+^ CD80^+^ CD86^+^ while in the center, they were uniformly immature phenotype MHC II^-^ CD80^-^ CD86^-^. More recently, Hattori et al. [9] described new langerin-expressing DCs that initiate adaptive immunity in normal corneas.

The local microenvironment has been widely recognized as an important regulator for APC maturation. Some cytokines, such as transforming growth factor beta (TGF-β), interleukin 10 (IL-10), prostaglandin E2 (PGE_2_), and the macrophage colony stimulating factor (M-CSF), have been recently employed to regulate DC maturation [10]. In addition, the ocular microenvironment is rich with immunosuppressive molecules that influence immune cells activity. For instance, TGF-β_2_, alpha-melanocyte-stimulating hormones (α-MSH), and calcitonin gene-related peptides (CGRP) in the aqueous humor can regulate the maturation of DCs [11].

Therefore, the factors that inhibit DC maturation, especially in terms of corneal transplant rejection, and the known roles of DCs in the development and persistence of some corneal diseases are important investigations [12]. Shen et al. [13] and our team [14] found that the aqueous humor prevents DC maturation through TGF-β_2_ in vitro. This study was performed to further investigate the cytokines involved in the regulation of corneal stroma cells (CSCs) in DC maturation.

## Methods

### Experimental animals

Six- to eight-week-old male BALB/c and C57BL/6 mice were purchased from Hebei Medical University Animals Science Research Center, Shijiazhuang, China. All experimental procedures were treated according to the ARVO Statement for the Use of Animals in Ophthalmic and Vision Research and approved by the Hebei Medical University Institutional Animal Care and Use Committee.

### Materials

RPMI 1640 medium, collagenase I, mitomycin C, lipopolysaccharide (LPS), Hoechst 33342, and Fluorescein isothiocyanate (FITC) -conjugated dextran were purchased from Sigma-Aldrich (St. Louis, MO). Ethylene diamine tetraacetic acid (EDTA) and TRIzol® reagent were purchased from Invitrogen-Gibco BRL (Grand Island, NY). Rabbit anti-mouse keratocan (clone H-50) and FITC-conjugated goat anti-rabbit immunoglobin G (IgG) were purchased from Santa Cruz (Santa Cruz, CA). Fetal bovine serum (FBS) was purchased from Hyclone (Logan, UT). A CellTiter 96® AQueous One Solution Cell Proliferation Assay (MTS) kit, Murine leukemia virus reverse transcriptase, and Taq DNA polymerase were purchased from Promega (Madison, NY). Nylon wool columns were purchased from Kisker (Postfach, Steinfurt, Germany). Rat anti-mouse CD16/32, polyethylene (PE)-conjugated Armenian hamster anti-mouse CD11c (clone N418), FITC-conjugated Armenian hamster anti-mouse CD80 (clone 16–10A1) and CD3 (clone 145–2C11), FITC-conjugated rat anti-mouse CD86 (clone GL1) and MHC II(clone M5/114.15.2), and the respective isotype-matched antibodies were purchased from eBioscience (San Diego, CA). Enzyme-linked immunosorbent assay (ELISA) kits to detect the cytokines TGF-β_2_, neutralizing TGF-β_2_ antibody (clone AB-112-NA), normal goat IgG, and recombinant murine GM-CSF (rmGM-CSF) were purchased from R&D Systems (Minneapolis, MN).

### Isolation of murine CSCs

CSCs were prepared from the cornea as previously described [15], with minor modifications. After euthanasia, the eyeballs were enucleated using forceps, washed three times with phosphate-buffered saline (PBS), and immersed in PBS containing 4000 U/ml gentamicin for 15 min. After rinsing with PBS, the central corneal button of each eyeball was cut off under the microscope. The corneal buttons were incubated in PBS containing 20 mmol/l EDTA for 45 min at 37 °C. Then, the epithelium was carefully removed from the corneal stroma by scraping the outer surface of the cornea while the corneal endothelium was peeled away from the inner surface of the cornea using fine forceps. After the peripheral cornea (including the limbal region) was removed, the corneal stromas were dissolved with 300 U/ml of collagenase I in RPMI 1640 at 37 °C. Four hours later, the isolated single cells were centrifuged and cultured in a serum-free RPMI 1640 medium (10^5^ CSCs/ml) at 37 °C in a 5% CO_2_ atmosphere. After 72 h, half the medium was changed. Another 72 h later, the supernatant was harvested and tested.

### Immunocytochemistry

The CSCs were cultured in serum-free RPMI 1640 medium for four to seven days. Cell cultures were washed with PBS, fixed in 4% paraformaldehyde/PBS for 10 to 15 min, and rinsed with PBS; then, they were permeabilized with 0.2% Triton X-100/PBS for 10 min and blocked in 3% bovine serum albumin (BSA) for 20 min. Samples were then incubated overnight at 4 °C with their primary antibodies diluted in 3% BSA/PBS (rabbit anti-keratocan, 1:200 dilution); control samples were incubated overnight at 4 °C with an isotype-matched nonimmune serum diluted in 3% BSA/PBS. After three PBS washes, all the samples were incubated 1 h at room temperature in the dark with their secondary antibodies diluted in 3% BSA/PBS (FITC-conjugated goat anti-rabbit IgG, 1:100 dilution). After washing the samples three times with PBS, they were counterstained with Hoechst 33342 for 1 min. Images were obtained using a fluorescence microscope (TS-100; Nikon, Tokyo, Japan).

### Reverse transcription polymerase chain reaction (RT–PCR)

Total ribonucleic acid (RNA) was extracted from primary murine CSCs using a TRIzol® reagent, according to the manufacturer’s instructions. A single-stranded complementary deoxyribonucleic acid (cDNA) copy, made from total RNA using a murine leukemia virus reverse transcriptase, was subjected to a semiquantitative PCR with primers (Table 1). The amplification was performed in a DNA thermal cycler (PX2; Thermo Fisher Scientific, Waltham, MA) as follows: initial denaturation at 94 °C for 5 min, denaturation at 94 °C for 30 s, annealing for 30 s, and extension at 72 °C for 45 s, for a total of 30 cycles, then a final extension at 72 °C for 10 min was performed. The PCR products were analyzed on a 2% agarose gel and scanned using an ultraviolet (UV) illuminator (Bio-Print; Vilber Lourmat, Marne-la-Vallée, France). The expression of various markers was normalized using β-actin (*ACTB*) as an internal control.

**Table 1:** PCR Primers.

**Gene**	**Primer sequence (5′→3′)**	**Product size (bp)**	**Annealing temperature**	**GenBank accession ID**
Keratocan	Forward: TCTGTCCTCCCAGTTTCC	205	50 °C	NM_008438
** **	Reverse: CCCTTCTCAATCCCGTAG	** **	** **	** **
*ALDH*	Forward: GGAGCACGGCTGTAGGAA	325	52 °C	NM_007436
** **	Reverse: TTTATGACCCGCTGGAAG	** **	** **	** **
*TGF-β_2_*	Forward: GGGGTTAAGGAGGTGG	229	57 °C	NM_009367
** **	Reverse: GGAGACTCGGTGGGATA	** **	** **	** **
*PTGS_2_*	Forward: CTCTGCGATGCTCTTC	268	53 °C	NM_011198
** **	Reverse: AATGTTCCAGACTCCCT	** **	** **	** **
*M-CSF*	Forward: ATGATGCCAAGAGGAAGACA	159	54 °C	NM_007778
** **	Reverse: CATAGGGATGGATGGGACA	** **	** **	** **
*IL-10*	Forward: ACCAAAGCCACAAAGCAG	283	54 °C	NM_010548
** **	Reverse: GGCAACCCAAGTAACCCT	** **	** **	** **
β-actin	Forward: GGAAATCGTGCGTGACATTA	379	54 °C	NM_007393
** **	Reverse: GGAGCAATGATCTTGATCTTC	** **	** **	** **

### Preparation of DCs from bone marrow

The isolation and culture of bone marrow-derived DCs were performed according to the established protocols [2]. Bone marrow mononuclear cells were prepared by C57BL/6 mouse femur bone marrow suspension throught the depletion of red cells, and then, they were cultured in 6-well plates in RPMI 1640 medium supplemented with 10% FBS and 10 ng/ml rmGM-CSF. The medium was changed on day two and half the medium was changed on day five. On day eight, nonadherent and loosely adherent cells were harvested as immature DCs. To induce DC maturation, LPS (1 μg/ml) was added, and the solution was allowed to culture for another 48 h. The purity of the DCs was determined using the flow analysis of surface CD11c staining.

### Analysis of the immunomodulatory factor

The levels of TGF-β_2_ in the CSC culture supernatant and fresh medium were measured using the ELISA kit (detection limit 7.0 pg/ml). To determine the effect of the CSC culture supernatant on DC maturation, during the maturation stage of DCs, various concentrations of the supernatant (25%, 50%) were added into the culture medium. To verify the immunomodulatory function of TGF-β_2_ in the CSC culture supernatant further, in parallel experiments (with 50% supernatant), a neutralizing TGF-β_2_ antibody of sufficient concentration (15 μg/ml) was added into the CSC culture supernatant. Normal goat IgG was used as a negative control.

### Endocytosis assay

Endocytosis was measured as the cellular uptake of FITC-dextran and was quantified using flow cytometry. Approximately 5×10^5^ cultured DCs per sample were incubated at 37 °C for 45 min in a medium containing FITC-dextran (1 mg/ml; molecular weight 40,000). Then, the cells were washed twice with cold PBS to stop endocytosis and remove excess dextran. The quantitative uptake of FITC-dextran was determined using flow cytometry analysis. A parallel experiment performed at 4 °C served as a negative control.

### Mixed leukocyte reactions (MLRs)

Splenic T cells from BALB/c mice were collected using nylon wool columns according to the manufacturer’s instructions. The purity of the cultured T cells was determined using the flow analysis of surface CD3 staining. After pretreatment with mitomycin C (30 μg/ml for 30 min at 37 °C), cultured DCs were seeded in triplicate at 2×10^4^, 1×10^4^, 4×10^3^, and 2×10^3^ cells per 200 μl/well in 96-well U-bottomed plates with 2×10^5^ allogeneic T cells. Three days later, 20 μl of an MTS reagent were added to each well, and the plates were incubated with MTS for 4 h at 37 °C. The light absorbent value (*A*) at a 490nm wavelength was measured using a microplate reader (Dynex Technologies, Billingshurst, UK). To correct the background activity, mediums with and without T cells served as controls. The stimulate index (SI) was calculated using the following formula: SI=(*A* value of experiment group - *A* value of medium control group)/(*A* value of T cells control group - *A* value of medium control group). The experiments were repeated in triplicate with at least three different cultured specimens.

### Flow cytometry analysis

DCs and T cells were harvested, washed twice in PBS, and resuspended in a fluorescence-activated cell sorter washing buffer (2% FBS and 0.1% sodium azide in PBS). After they are blocked for 15 min at 4 °C with a CD16/32 antibody, the cells were incubated for 40 min at 4 °C in the dark with their respective fluorescent antibodies. For DCs, the following monoclonal antibodies were used: CD11c-PE, CD80-FITC, CD86-FITC, and MHC II-FITC; for T cells, the CD3-FITC antibody was applied. Their respective isotype-matched antibodies served as negative controls. A total of 2×10^4^ cell events were analyzed on the flow cytometer (FACS; BD Biosciences PharMingen, San Diego, CA) by gating out the majority of non-viable cells based on low forward angle light scatter.

### Statistical analysis

Statistical analysis was performed by using SPSS 13.0 (SPSS Inc., Chicago, IL). Data represent mean value±standard deviation. Statistical significance was evaluated using the paired student’s *t*-test and a p-value <0.05 was considered statistically significant.

## Results

### CSCs, T cells, and DCs were isolated

**Figure 1:**
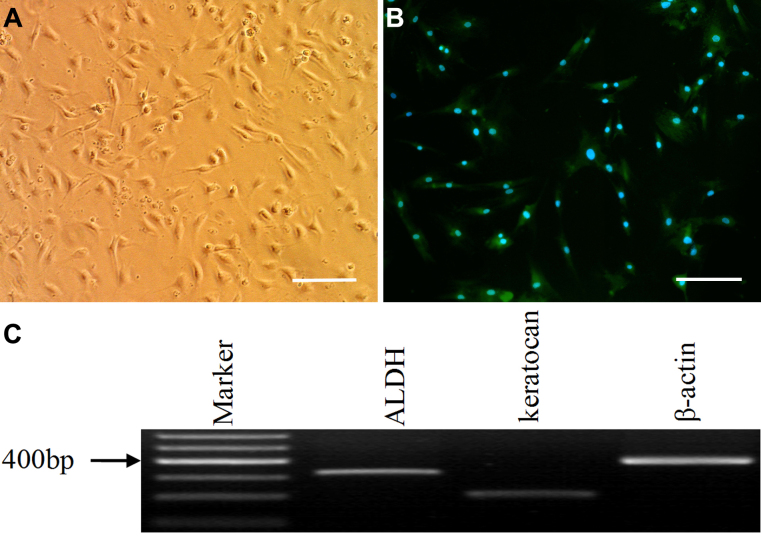
Isolated murine CSCs. CSCs exhibited condensed cell bodies with numerous processes (**A**), positive immunofluorescence staining of keratocan protein (**B**), positive expression of *ALDH* and keratocan genes. Bar=35 μm.

First, different types of cells were prepared and identified. For CSCs, after corneal stromas were dissolved using collagenase, single cells were obtained. Within four hours after seeding the cells in a serum-free RPMI 1640 medium, they were attached to the plastic and exhibited condensed cell bodies with numerous branching processes radiating from each cell (Figure 1A), and they maintained the protein expression of keratocan (Figure 1B) and the gene expression of aldehyde dehydrogenase (*ALDH*) and keratocan (Figure 1C).

**Figure 2:**
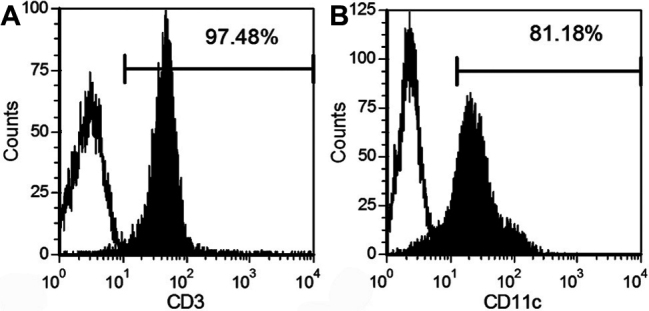
The phenotypic characterization of cultured cells by flow cytometry analysis. Data demonstrated that T cells were positive for CD3 (**A**), and DCs were positive for CD11c (**B**). Isotype control (open histogram) and stain with relevant antibody (filled histogram) are shown.

**Figure 3:**
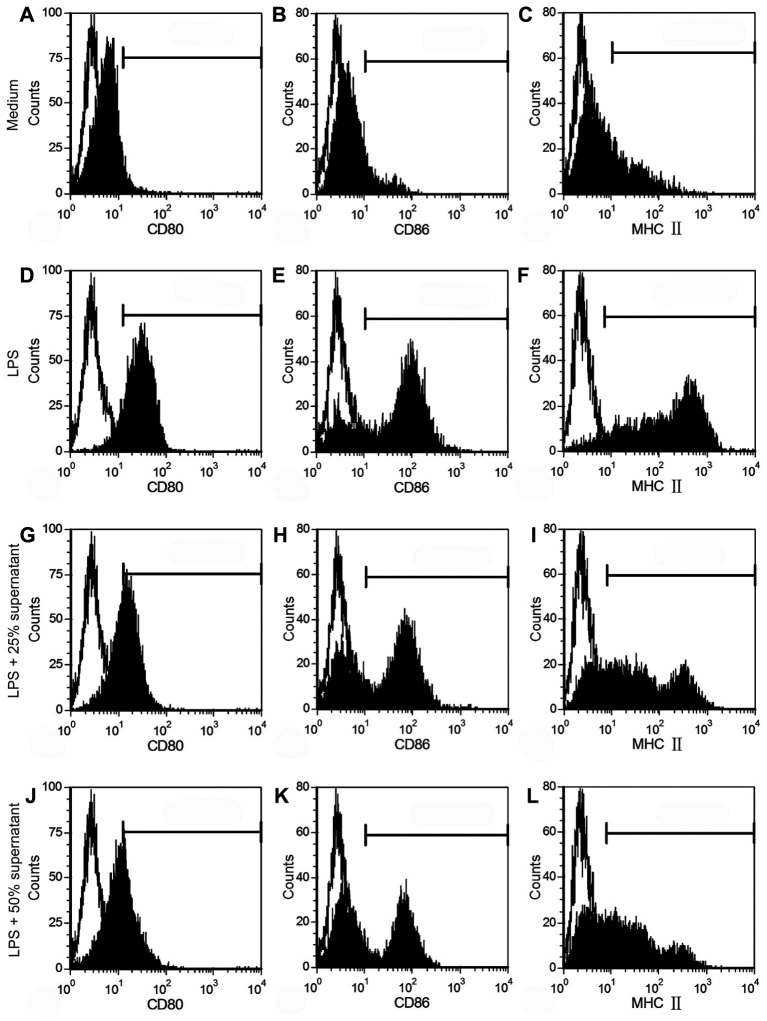
Flow cytometry analysis demonstrated that CSC culture supernatant suppressed the expression of costimulatory molecules, CD80 and CD86, and MHC II in a dose-dependent manner during DC maturation. The expression of each surface molecule (filled) compared to the respective isotype control (empty). Representative example of three experiments.

For T cells, following the depletion of the red cells, splenic cells were passed through nylon wool columns, and nonadherent cells were harvested. Phenotypic analysis using flow cytometry indicated that these cells were positive for CD3 (93.97%±3.06%, Figure 2A), which is considered a hallmark for T cells. For DCs, three days after the addition of rmGM-CSF to the culture medium, bone marrow mononuclear cells generated many distinctive cell clusters that attached to the plate bottoms. On the eighth days of the culture, immature bone marrow-derived DCs expressed high levels of CD11c (78.61%±4.27%, Figure 2B) but low levels of the maturation markers MHC II, CD80, and CD86 (Figure 3A-C). After an additional two days of LPS stimulation, mature DCs were harvested. As expected, mature DCs became non-adherent and displayed different protruding veils with abundant cytoplasm, which is a typical morphology for DCs. In parallel, matured DCs were accompanied by increased expressions of the maturation markers (Figure 3D-F, Table 2) and the capability to stimulate the proliferation of T lymphocytes (Table 3). These data demonstrate that we have isolated T cells and DCs successfully in the present experiment.

**Table 2:** Expression of CD11c, CD80, CD86, MHC II, and dextran on DCs.

** **	**DCs treatment**				
**Index**	**immature**	**LPS**	**LPS + 25% supernatant**	**LPS + 50% supernatant**	**LPS + 50% supernatant + TGF-β2 antibody**
CD80	18.62±3.66	94.17±6.74	61.44±4.76^A^	48.75±4.69^AB^	65.21±4.76^C^
CD86	48.06±7.60	177.05±12.52	131.94±10.00^A^	107.22±9.78^AB^	133.56±8.63^C^
MHC II	91.92±11.29	253.10±10.43	191.73±19.35^A^	151.77±13.94^AB^	184.28±9.29^C^
dextran	―	347.51±14.42	387.10±10.73^A^	439.40±21.46^AB^	390.23±12.67^C^
CD11c	50.40±3.20	49.79±4.12	51.15±3.80^NS^	48.49±4.95^NS^	49.52±4.16^NS^

Data represent mean value±standard deviation of mean fluorescence intensity (MFI). ^A^, p<0.05 compared with DCs treated with LPS; ^B^, p<0.05 compared with DCs treated with LPS + 25% CSCs culture supernatant; ^C^, p<0.05 compared with DCs treated with LPS + 50% CSCs culture supernatant; ^NS^, no significance.

**Table 3:** Allogeneic T lymphocyte stimulatory capacity of DCs.

** **	**DCs treatment**			
**DCs/T cell ratio**	**LPS**	**LPS + 25% supernatant**	**LPS + 50% supernatant**	**LPS + 50% supernatant + TGF-β2 antibody**
1/10	11.57±0.54	8.16±0.46^A^	4.83±0.31^AB^	7.84±0.45^C^
1/20	6.81±0.32	7.47±0.39^A^	3.02±0.29^AB^	4.82±0.28^C^
1/50	3.96±0.40	3.03±0.31^A^	1.95±0.23^AB^	2.74±0.25^C^
1/100	2.32±0.27	1.80±0.28^NS^	1.23±0.24^ANS^	1.63±0.22^C^

Data represent mean value±standard deviation of stimulate index (SI). ^A^, p<0.05 compared with DCs treated with LPS; ^B^, p<0.05 compared with DCs treated with LPS + 25% CSCs culture supernatant; ^C^, p<0.05 compared with DCs treated with LPS + 50% CSCs culture supernatant; ^NS^, no significance.

### CSC culture supernatant suppresses DC phenotypic and functional maturation

In the next step, we investigated the effect of the CSC culture supernatant on DC maturation. On day eight of the DC culture, the supernatant was added into the culture medium. As presented in Figure 3G-I, the treatment of immature DCs with the CSC culture supernatant, though LPS supplemention, caused a remarkable reduction in the maturation markers, CD80, CD86, and MHC II (Table 2). Furthermore, the suppressive effect seemed to be supernatant dose-dependent. However, the expression of CD11c was not altered (Table 2), which indicates that the changes of DC maturation markers were not due to cell death or a general downregulation of protein synthesis.

**Figure 4:**
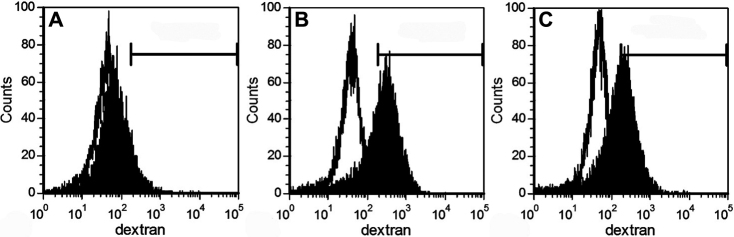
Flow cytometric analysis of endocytic activity of DCs. Mature DCs (**A**), DCs treated with 50% CSC culture supernatant (**B**), and DCs treated with 50% CSC culture supernatant in presence of neutralizing TGF-β2 antibody (**C**). The empty histograms represent the uptake of FITC-dextran at 4 °C. The histogram is from one representative experiment out of three performed.

The suppression of the DC phenotypic maturation using the CSC culture supernatant led us to further investigate its effect on the modulation of DC functions. The ability of DCs to induce T cell proliferation in the MLRs assay is commonly used for functional evaluation. In the current study, the results of the MLRs showed that the CSC culture supernatant treatment resulted in a dose-dependent reduction in the efficiency of DC-stimulated allogeneic T cells (Table 3). Immature DCs possess stronger antigen uptake activity than mature DCs. Therefore, we tested the endocytosis of FITC-dextran as a measurement of antigen uptake. We found that with the addition of 50% supernatant, treated DCs exhibited clearly higher endocytic activity than untreated DCs (Figure 4A,B, Table 2).

### CSCs inhibit DC maturation via TGF-β_2_ secretion

**Figure 5:**
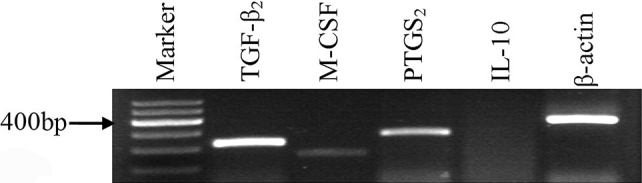
RT–PCR analysis of immunomodulatory cytokines in CSCs. CSCs showed positive expression of *TGF-β_2_*, *PTGS_2_*, and *M-CSF*, but negative expression of *IL-10*.

Following our observation that the CSC culture supernatant inhibited DC maturation, we evaluated which immunomodulatory cytokine secreted by CSCs is involved in the immunosuppressive process. Previous reports indicated that some soluble factors are essential in the molecular control of DC development. Therefore, we attempted to identify the gene expression of several immunomodulatory cytokines, including TGF-β_2_, M-CSF, and IL-10, and the expression of prostaglandin endoperoxide synthase 2 (PTGS_2_, a key enzyme in the biosynthesis of PGE_2_). As shown in Figure 5, CSCs exhibited positive expressions of TGF-β_2_, PTGS_2_, and M-CSF, but a negative expression of IL-10. Furthermore, the quantitative analysis of cytokines production using ELISA showed a higher concentration of TGF-β_2_ in CSC culture supernatant (1.46±0.38 ng/ml) than in the fresh medium (0.11±0.06 ng/ml), which was consistent with the results of RT–PCR.

**Figure 6:**
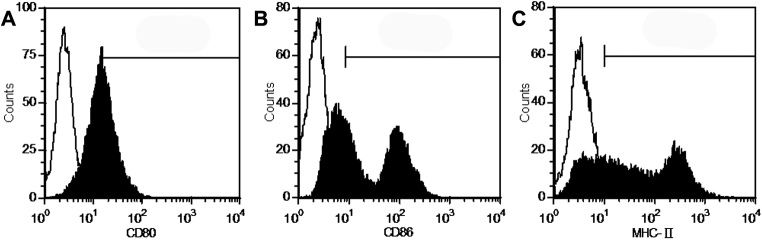
The phenotypic characterization of DCs treated with 50% CSC culture supernatant in presence of neutralizing TGF-β_2_ antibody, measured by flow cytometry. The expression of each surface molecule (filled) compared to the respective isotype control (empty). Representative example of three experiments.

As TGF-β_2_ was detected in the CSC culture supernatant, in the next experiment, we examined whether inhibiting its functions could reverse the supernatant-mediated immunomodulatory effects. After adding the saturating concentrations of the neutralizing TGF-β_2_ antibody (15 μg/ml, 10,000 times of TGF-β_2_ concentration in CSC culture supernatant) into the CSC culture supernatant, the phenotypic modifications induced by the supernatant were modestly reversed (Figure 6, Table 2). Furthermore, in the presence of the antibody, there was a significant increase in T cell stimulation capacity (Table 3) and a notable decrease in endocytic activity (Figure 4C, Table 2).

## Discussion

Recent evidence has demonstrated that the cornea contains a heterogeneous population of bone marrow-derived cells, including epithelial langerhans cells and stromal DCs, which can function as APCs under certain conditions. While the peripheral cornea contains mature and immature resident bone marrow-derived DCs, the central cornea is endowed exclusively with highly immature/precursor-type DCs [16,17]. We speculated that DCs in the central cornea could be kept in an immature state due to the suppressive effect of some cornea-derived factors. Therefore, in this study, we investigated the effect of CSC culture supernatant on the maturation of bone marrow-derived DCs.

Earlier studies used dendritic morphology and extensive intercellular contacts as the hallmarks for the keratocyte phenotype [18]. Subsequently, other markers for keratocytes were reported, including keratan sulfate-containing proteoglycans, such as keratocan [19] and ALDH [20]. In our study, the protein expression of keratocan and the gene expressions of *ALDH* and keratocan were detected in CSCs. Moreover, in our previous study, we found that these cells did not express markers of monocyte-derived cells-CD34 and CD45 [21].The morphology of single cells, similar to that of keratocytes in situ, and the expression of stromal markers in these cells showed that the CSCs in our study were of stromal origin.

Then, we found that the CSC culture supernatant could down-regulate CD80, CD86, and MHC II expressions on DC surfaces, inhibit the MLRs, and upregulate the antigen uptake ability in DCs. These effects seemed to be CSC culture supernatant dose-dependent. Furthermore, compared with our previous research results, adding DMSO and normal goat IgG into the DCs culture medium did not change expression of specific DCs markers, including CD11c, CD80, CD86, and MHC II (data not shown). Therefore, in the supernatant, soluble immunosuppressive cytokines must be secreted by the CSCs.

In a recent study, Shen et al. [13] incubated corneas in medium for 48 h, collected the supernatant, and then applied the corneal supernatant during TNF-α-induced dendritic cell maturation. The authors found that the corneal supernatant promoted the generation of phenotypically and functionally immature DCs due in part to TGF-β_2_ but not to α-MSH, CGRP and TGF-β_1_. However, the cytokine TGF-β_2_ can diffuse from the aqueous humor into the cornea, and the corneal epithelium also can also produce TGF-β_2_ [22].

Previous studies indicate that when they are cultured in a complete medium, CSCs readily lost their phenotype, transformed into fibroblast or myofibroblast, and expressed *TGF-β_2_* mRNA [23,24]. In addition, the serum contains many cytokines, including TGF-β, and the cytokine concentration varies even if it is from the same company. To avoid possible confusion, we isolated and cultured the CSCs in a serum-free culture medium and changed half the medium every 72 h. Moreover, we believe that this culture system has potential to contribute to future discoveries and is a good in vitro model that can be used to study the role of the factors secreted by CSCs in DC maturation, which is worth investigating.

In the present study, we found positive expressions of PTGS_2_ and TGF-β_2_ in the CSCs, and high concentration of TGF-β_2_ (1.46±0.38 ng/ml) and PGE_2_ (data not shown) in the supernatant [25]. To determine whether the immunosuppressive activity of the supernatant is due to its content of TGF-β_2_ and to examine whether this TGF-β_2_-mediated inhibition of DC maturation is limited to TNF-α-stimulated maturation [13], a neutralizing TGF-β_2_ antibody was applied during the DCs’ LPS-induced maturation stage. As a result, phenotypic and functional modifications of DCs induced by the supernatant were modestly reversed. The present findings suggest that CSCs can inhibit LPS-induced DC maturity through the secretion of TGF-β_2_.

TGF-β_2_, a highly conserved, homodimeric protein, has been intensively studied for its immunoregulatory capacity. Various studies have demonstrated that after TGF-β_2_ treatment, DCs are resistant to maturation, and naive T cells are further polarized toward regulatory T cells [26]. It has been demonstrated that the epithelium of the airway and gut have inhibitory effects on DCs through the spontaneous and constitutive secretion of TGF-β, which as a mechanism of peripheral tolerance [27,28]. Similarly, in the present study, CSCs inhibit DC maturation through TGF-β_2_ secretion. Thus, we believe that this finding may enrich the interpretive mode of corneal immune privilege.

Another immune mediator, PGE_2_, can be produced in various types of cells. In fact, CSCs could secrete PGE_2_ in vitro. However, compared with TGF-β_2_, the PGE_2_ present in the supernatant exhibited a paradoxical effect on DCs [25]. Other key factors that alter the maturation process of DCs are IL-10 and M-CSF. Their DC immunosuppressive properties result in a reduced expression of MHC II molecules as well as costimulatory and adhesion molecules [29,30]. In our study, negative expressions of IL-10 suggest that cytokine is unlikely to be involved in the CSCs-derived inhibition of DCs in vitro.

Because of its direct exposure to the external environment, the cornea has to develop sophisticated defense mechanisms to protect itself [31]. Due to their great endocytosis ability, immature DCs in the central cornea are ideally suited to clear trivial antigens. Therefore, we presume that the exclusive presence of immature DCs in the central cornea ensures the maximum clearance of foreign antigens without inducing blinding inflammatory damage. When detrimental pathogens invade, the release of proinflammatory cytokines disrupts ocular homeostasis and creates a microenvironment that activates immature DCs. Previous studies have demonstrated that a subset of MHC II-negative stromal DCs residing in the center of the cornea can significantly upregulate the expression of MHC II as early as 24 h after the initiation of inflammation [32]. In addition, during inflammation, the expressions of CD80, CD86, and CD40 increase [33].

Nevertheless, due to the accelerated and enhanced delayed-type hypersensitivity response induced by mature DCs, the number of mature DCs present in the central cornea has been shown to correlate with the degree of corneal damage [34]. Similar to the inflammatory disorders, the maturation stage of corneal DCs in autoimmune keratopathies has also been proved abnormal [35]. Furthermore, data indicate that mature host cornea-derived DCs can present graft antigens to host T cells and cause corneal allograft rejection in corneal transplantation [36].

However, some problems need to be further explored. First, in this study, we detected that CSCs cultured in medium can secrete some cytokines in vitro. Whether these cells from the intravital cornea have the same capacity remains unclear. Accordingly, our assays could not fully confirm that intravital CSCs secret TGF-β_2_ and do not secret IL-10 or M-CSF. Second, it has been proven that the CSC culture supernatant can inhibit the maturation of DCs, and the cytokine TGF-β_2_ is in the activation state. Whether the TGF-β_2_ secreted by the CSCs needs to be activated from a latent state should be examined. In addition to TGF-β_2_, other immunosuppressive cytokines in the supernatant also need to be studied. Third, in this study, to avoid possible confusion with other types cells in peripheral cornea, such as limbal stem cells, we obtained our CSCs from the central corneal stroma. Whether CSCs from the peripheral cornea have the same immunomodulatory action should be investigated. Fourth, in the peripheral cornea DCs are mature or immature, but in the central cornea they are exclusively immature. We concede that there may be possible reasons to explain this phenomenon; for instance: there is an increasing concentration gradient of suppressive factors from the peripheral cornea to the central cornea, secreted by CSCs, such as TGF-β_2_, diffusing from the center of the cornea; maturation factors produced by the peripheral corneal cells or conjunctival cells promote the maturity of DCs in the periphery of the cornea; and the DC maturation function of CSCs from the peripheral and central cornea may be different. These possibilities should be examined in the future studies.

In conclusion, the present study has identified that CSCs can partially inhibit LPS-induced DC maturation through TGF-β_2_ secretion in vitro. Considering the critical role of DCs in all ocular immune responses, further studies on the regulation of DC maturation should be considered. Furthermore, we believe that a better understanding of the molecular structures that lead to DC immaturation in the cornea will certainly provide new approaches to corneal transplantation and the treatment of corneal autoimmune or inflammatory diseases.
